# Decomposition Characteristics of the TTIP (Tetraisopropyl Orthotitanate) Precursor for Atomic Layer Deposition

**DOI:** 10.3390/ma15093021

**Published:** 2022-04-21

**Authors:** Hayeong Kim, Jihyeok An, SeonJeong Maeng, Jae-Soo Shin, Eunmi Choi, Ju-Young Yun

**Affiliations:** 1 Vacuum Materials Measurement Team, Korea Research Institute of Standards and Science, Daejeon 34113, Korea; hykim1008@kriss.re.kr (H.K.); sjmaeng14@kriss.re.kr (S.M.); 2 Nanomaterials Science and Engineering, University of Science and Technology, Daejeon 34113, Korea; 3 Process Development Group, Semiconductor Division/R&D Center, DUKSAN Techopia Co., Ltd., Cheonan 31245, Korea; jhan@oneduksan.com; 4 Department of Energy & Advanced Materials Engineering, Daejeon University, Daejeon 34520, Korea; jsshin@dju.ac.kr; 5 Defect Engineering Group, SK hynix, Icheon 17336, Korea; 6 Nanoscience and Technology, University of Science and Technology, Daejeon 34113, Korea

**Keywords:** atomic layer deposition (ALD), titanium dioxide (TiO_2_), precursor, tetraisopropyl orthotitanate, thermal decomposition

## Abstract

The decomposition of tetraisopropyl orthotitanate (TTIP), a representative precursor used in the atomic layer deposition (ALD) of titanium dioxide (TiO_2_) film, and the resulting changes in the thin film properties of the TiO_2_ film were investigated. TTIP was evaluated after exposure to thermal stress in an enclosed container. The vapor pressure results provide reasonable evidence that impurities are generated by the decomposition of TTIP under thermal stress. These impurities led to changes in the thermal properties of TTIP and changes in the growth rate, morphology, and composition of the thin film; in particular, these impurities increased the unstable oxidation states of Ti^2+^ (TiO) content in the TiO_2_ film. The changes in the properties of the TiO_2_ film resulting from the changes in the physical properties of TTIP led to a change in the properties of the device. We proved that the thermal stability of the precursor is a factor that can determine the reliability of the ALD process and the resulting thin film. Additionally, systematic evaluation of the precursor can provide useful information that can improve the development of the precursor and the consistency of the process.

## 1. Introduction

Recently, capacitance has decreased due to the high integration of devices because the area occupied by the metal–oxide–semiconductor (MOS) capacitor, which serves to store data, is reduced as devices are increasingly integrated to increase performance [1,2,3]. To solve this problem, the thickness of the SiO_2_ used as the dielectric layer of the MOS capacitor is reduced to reach appropriate levels of capacitance. However, there is a limit to how much the SiO_2_ thickness can be reduced, because critical issues such as leakage current can occur if the thickness of SiO_2_ decreases to a certain level [4,5]. High electrical permittivity (high-k) materials that can replace SiO_2_, such as HfO_2_ [6,7], Al_2_O_3_ [8], ZrO_2_ [9,10], La_2_O_3_ [11], Ta_2_O_5_ [12,13], and TiO_2_ [14], have been widely investigated for the next-generation of MOS capacitors. Specifically, TiO_2_ has attracted attention as a next-generation high-k material due to its desirable characteristics, including a high dielectric constant (anatase~19, rutile~86), low leakage current density, and high thermal stability [15,16,17].

Various deposition techniques such as atomic layer deposition (ALD), pulsed laser deposition (PLD), sol–gel, sputtering, etc., can be applied to TiO_2_ thin film deposition [18]. Among them, ALD, a deposition technique based on self-limiting surface chemistry, is used to successfully deposit TiO_2_ thin films because of several advantages, including low process temperature, excellent step coverage, thickness control at the atomic level, and excellent uniformity [19,20]. To achieve the ideal ALD process, the precursor should become saturated after a self-limiting reaction with the surface, and unnecessary impurities such as ligands and hydroxyl groups must be completely removed by a purge gas after a complete reaction with the reactant [21]. The precursor should not only have high volatility to offer sufficient reproducibility, but it should also not cause an intra- or intermolecular reaction [22]. To satisfy the above characteristics, precursors for depositing TiO_2_ thin films have been designed by employing various ligands, such as halides, alkylamides, alkoxides, cyclopentadienyls, and heteroleptics [23]. However, most of the precursors are not self-limiting because exposure to thermal stress during storage and ALD processes causes decomposition due to intra- or intermolecular reactions. Additionally, these decomposition reactions not only hinder self-limiting growth and lower the uniformity of the thin film but also result in a high concentration of impurities [22,24].

The thermal stability of a precursor must be discussed for a successful ALD process, and many studies have already been carried out [25,26,27,28]. However, most of the studies related to the thermal stability of precursors have measured the decomposition temperature using thermogravimetric analysis (TGA) or studied the decomposition behavior in the gas phase by using quadrupole mass spectrometry (QMS) and a quartz crystal microbalance (QCM) [29,30,31]. Furthermore, S. Rushworth et al. reported the long-term thermal stability of three types of hafnium and zirconium precursors using nuclear magnetic resonance (NMR) [25]. However, the exposure time to thermal stress was as short as 50 h for all but one kind of precursor.

Nevertheless, the decomposition of a precursor occurs due to exposure to continuous thermal stress during precursor storage and transportation as well as vaporization in a canister. In addition, under thermal stress, the generated decomposition products can change the properties of a precursor, such as the volatility and viscosity, and have a direct effect on the decreased reliability of a thin film deposited by ALD [32,33]. Therefore, the long-term thermal stability of precursors must be evaluated rigorously. However, studies on the long-term stability of precursors and changes in the properties of precursors and thin films due to impurities generated by thermal stress have not been evaluated.

Therefore, in this study, the long-term thermal stability of a precursor, titanium isopropoxide (TTIP, Ti[OCH(CH_3_)_2_]_4_), which is one of the representative TiO_2_ precursors, was evaluated under exposure to thermal stress for 7 days under severe conditions (120~180 °C) using a newly made stainless steel container. The effect of TTIP degradation on the crystallography and composition of a thin film prepared through ALD was studied, an MOS capacitor device was fabricated, and the electrical characteristics were analyzed through C-V (capacitance–voltage) and I-V (current–voltage) measurements to analyze the effect of changes in the characteristics of the device. The relationship between the thermal stability of the precursor and the reliability of the device is discussed. Understanding the thermal decomposition of the precursor provides very useful information to achieve a successful ALD process.

## 2. Materials and Methods

**Figure 1:**
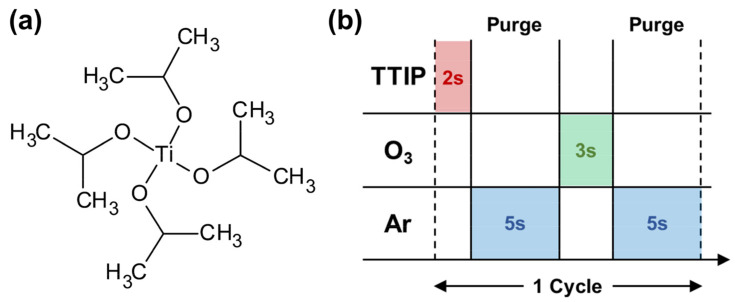
(**a**) Structure formula of titanium isopropoxide (TTIP) and (**b**) scheme of the atomic layer deposition process.

To investigate the correlation between the changes in the characteristics of the precursor and the changes in the characteristics of the thin film, including the device, due to deterioration, TTIP (Sigma Aldrich, 99.999% trace metals basis) (Figure 1a) was deteriorated in a furnace at 120, 150, and 180 °C for 7 days. In this process, we made a stainless steel container for heating the precursor, and VCR (vacuum coupling radiation) fittings were applied to the end of the container to prevent the precursor from exposure to the environment outside of the container. The VCR fitting was a metal gasket face seal-fitting type and prevented the precursor from reacting with moisture (H_2_O) and oxygen (O_2_), while precursors deteriorated in the furnace. TTIP was sampled in the stainless steel container in a glove box that maintained a Nitrogen (N_2_) atmosphere with moisture (H_2_O) and oxygen (O_2_) concentrations of 1 ppm or less.

TTIPs exposed to thermal stress under severe temperature conditions were analyzed using vapor pressure (homemade vapor pressure measuring apparatus) and thermogravimetric analysis (TGA, NETZSCH, STA 449 F3) to confirm the decomposition of TTIP.

The deteriorated TTIPs were then deposited on a p-type (100) wafer as a TiO_2_ thin film using ALD. Before the ALD process, the wafer was sonicated in acetone, ethanol, deionized (DI) water, and isopropyl alcohol (IPA) for 10 min. The pressure of the ALD chamber was 300 mTorr, and the substrate temperature was maintained at 250 °C. The temperature of the canister was set to 50 °C, and the line and shower head were set to 120 °C and 180 °C, respectively, gradually increasing the temperature to prevent condensation of the precursor. Ozone(O_3_) was used as the reactant gas; the gas was generated using an ozone generator (IN USA, Inc., OG-5000) by mixing O_2_ (99.999%) and Ar (99.999%). Figure 1b is the sequence of the already optimized ALD process, as follows: (1) source (gas phase precursor) feeding: 2 s; (2) purging with Ar gas: 5 s; (3) reactant (O_3_) feeding: 3 s; (4) purging with Ar gas 5 s. The ALD process was repeated for 500 cycles under fixed process conditions.

TiO_2_ thin films that were deposited using degraded TTIPs were analyzed using X-ray diffraction (XRD, RIGAKU, D/MAX-2500), X-ray photoelectron spectroscopy (XPS, Thermo Fisher Scientific, K-Alpha+, Waltham, MA, USA), and atomic force microscopy (AFM, Park Systems Corp., XE7, Suwon, Korea).

MOS capacitors were fabricated to measure the electrical properties of TiO_2_ thin films. The top electrodes were formed by patterning a 100 × 100 μm square pad using photolithography and lift-off techniques and depositing 200 nm of aluminum (Al) via thermal evaporation. Capacitance–voltage (C-V) measurements were performed using a precision LCR Meter (Agilent, HP4284A, Santa Clara, KA, USA) at 1 MHz in the range of −2 to +2 V. Current–voltage (I-V) measurements were measured using a semiconductor parameter analyzer (Agilent, HP 4155B) in the range of −2 to 2 V.

## 3. Results and Discussion

The decomposition of a precursor can be confirmed using ^1^H NMR [25], QMS [30], etc.; however, the presence or absence of the decomposition of a precursor can also be confirmed through vapor pressure measurement. This is because decomposition products generated by thermal stress change both the decomposition temperature and thermophysical properties, such as vapor pressure.

**Figure 2:**
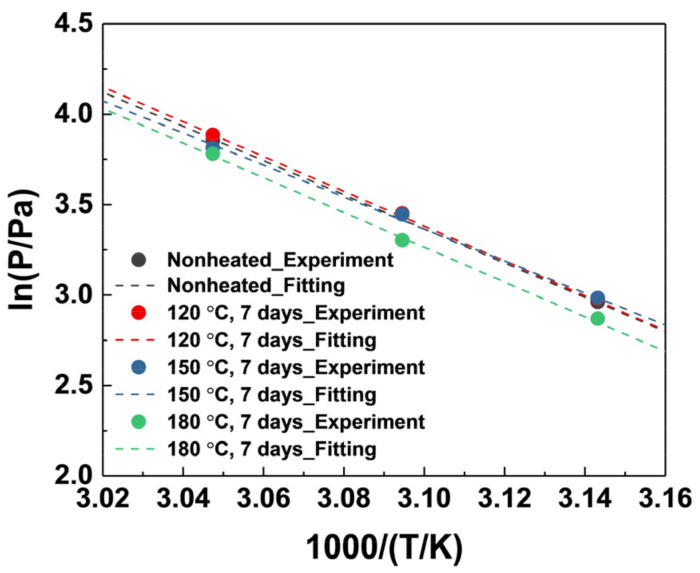
Comparison of the vapor pressure for TTIP exposed to thermal stress. The solid circles are the experimental vapor pressure value, and the dash lines represent the fitting of the Antoine equation corresponding to the experimental vapor pressure values.

Figure 2 shows the vapor pressure (at 45 °C, 50 °C and 55 °C) of degraded TTIP, and Table 1 lists the Antoine equation parameter and vaporization enthalpy $\left(∆H\right)$ at each temperature (vapor pressure measurement results are described in Supplementary Material Table S1). Vapor pressure was measured using the static method, and the vapor pressure curve was expressed using the Antoine equation (Equation (1)), as this is the most widely used correlation method for the extrapolation of vapor pressure in a range other than the measured temperature. The vaporization enthalpy was calculated using the Clausius–Clapeyron equation (Equation (2)) [34].

(1) $$\mathrm{lnP}\;=\;A\;-\frac{B}{T}$$

(2) $$\mathrm{lnP}\;=\;A\;-\frac{∆H}{RT}$$

According to the study of B. Blackburn et al., when TTIP is exposed to thermal stress, C-C bonds, O-C bonds, and Ti-O bonds break, leading to the generation of methyl, propyl, and isopropyl groups [35]. Therefore, it can be predicted that the generated methyl group is combined with the surrounding H atom to form methane, or other generated unstable molecules combine to form heavy molecules.

Considering the vapor pressure results, the vapor pressure at all of the measured temperatures of TTIP exposed to thermal stress of 120 °C slightly increases compared to TTIP not exposed to thermal stress, whereas the TTIP exposed to thermal stress of 180 °C decreases. As previously expected, light molecules such as methane exist as major decomposition products in TTIP exposed to a relatively low temperature (120 °C) for a long time, which leads to a rise in vapor pressure. Heavy molecules exist as major decomposition products when TTIP is exposed to high temperatures (180 °C), which leads to a decrease in vapor pressure. Thus, the vaporization enthalpy, a quantitative indicator of the volatilization properties of the precursor, also changes [36]. Therefore, if the volatilization properties are changed due to decomposition, the process of deposition may be changed, and consequently, the properties of the thin film may be affected. This is discussed in the properties of the thin film part of this paper.

**Figure 3:**
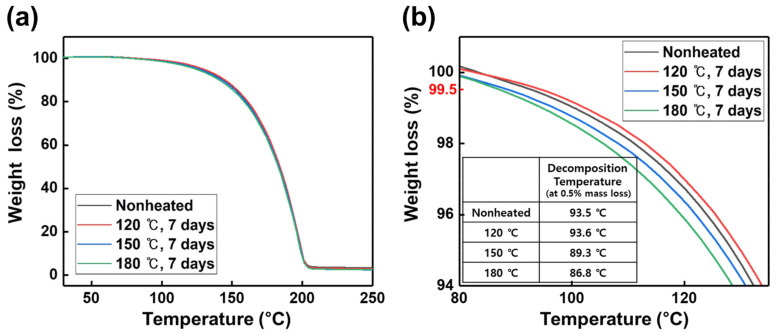
TG curves of TTIP exposed to thermal stress with a heating rate of 10 K/min under N_2_ atmosphere. (**a**) Full temperature range, (**b**) 80~150 °C range (related to the decomposition of TTIP).

Changes in TTIP properties due to the decomposition products of TTIP generated by thermal stress can also be confirmed through TGA. In general, the decomposition temperature of the precursor is defined as the temperature at which 0.5% mass loss of the precursor occurs [25]. Figure 3 shows the results of the TGA that was performed to confirm the decomposition temperature of TTIP exposed to thermal stress. The 0.5% mass loss of TTIP not exposed to thermal stress occurs at 93.5 °C, but when TTIP is exposed to thermal stresses of 120 °C, 150 °C, and 180 °C, the 0.5% mass loss occurs at 93.6 °C, 89.3 °C, and 86.8 °C, respectively. TTIP exposed to 120 °C thermal stress has a decomposition temperature similar to that of TTIP not exposed to thermal stress.

**Figure 4:**
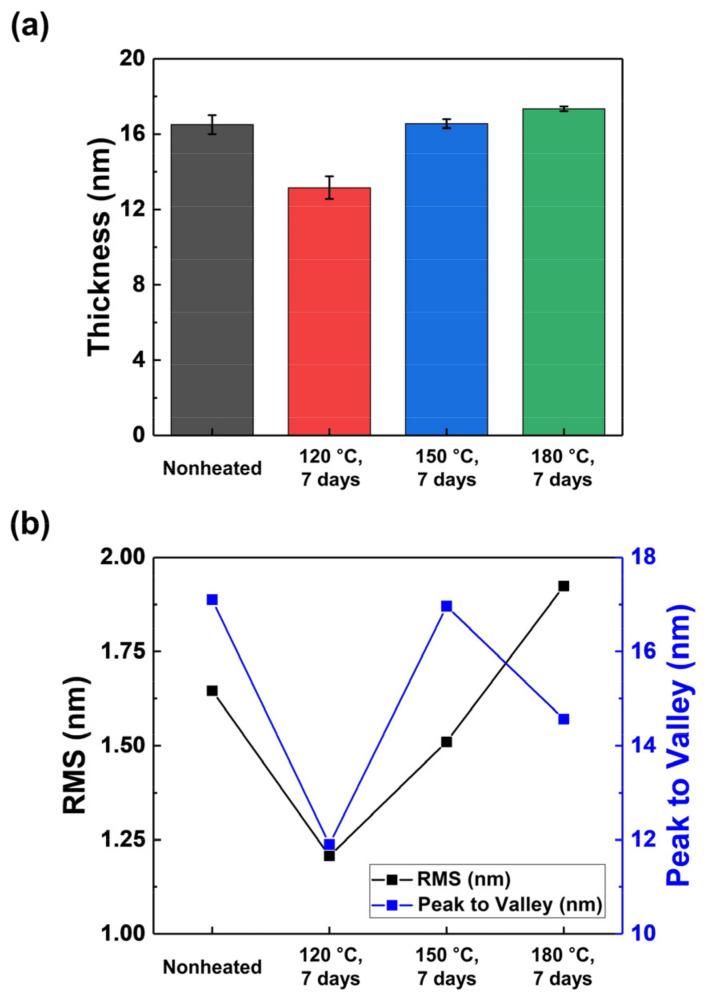
Properties of TiO_2_ thin films deposited by TTIP exposed to thermal stress. (**a**) Thickness of TiO_2_ thin films measured using ellipsometry, (**b**) RMS and peak to valley of TiO_2_ thin films determined using AFM.

Figure 4 shows the thickness and planarization information of TiO_2_ thin films grown in the same ALD process cycle using precursors exposed to thermal stress. As described above, it can be confirmed that the properties of thin films change because the properties of the precursor are changed. TTIP exposed to thermal stress of 180 °C showed an advantageous ALD reaction, indicating that the growth rate of the thin film is clearly improved. However, it can be confirmed that a thin film with low uniformity resulted from nucleation caused by heavy molecule impurities that induce a high thin film growth rate. On the other hand, the TiO_2_ thin film deposited by TTIP exposed to thermal stress of 120 °C has a low thin film growth rate despite having high vapor pressure. As described above, the vapor pressure is increased by light molecules such as methane, which are highly volatile, but the rate of growth of the thin film is decreased because the volatilization of the desired precursor molecules participating in the thin film growth is reduced.

**Figure 5:**
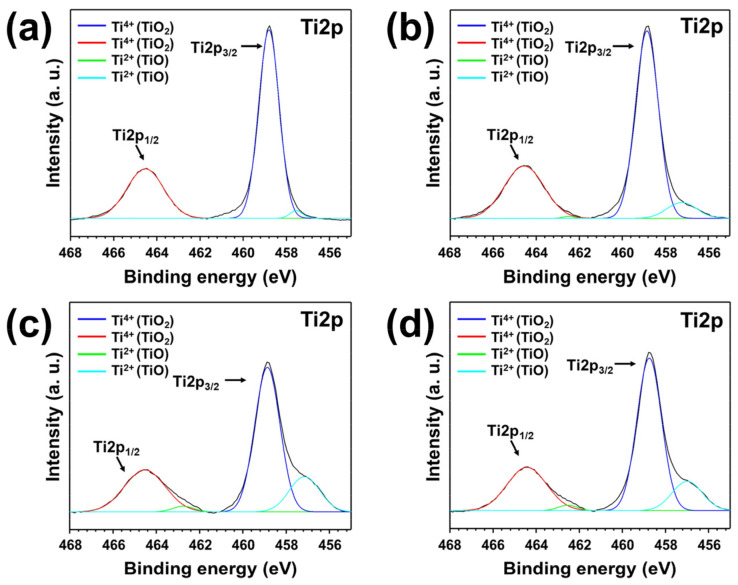
Ti2p XPS spectrum of TiO_2_ thin films deposited by TTIP exposed to thermal stress. (**a**) Nonheated TTIP, (**b**) TTIP exposed to 120 °C for 7 days, (**c**) TTIP exposed to 150 °C for 7 days, (**d**) TTIP exposed to 180 °C for 7 days.

Impurities due to the deterioration of TTIP affect the composition and crystallinity of the film, as well as its morphology. Figure 5 shows the XPS results for the TiO_2_ film deposited by TTIP exposed to thermal stress. Peak shifts at approximately 458.4 eV and 464.2 eV in Ti2p_3/2_ and Ti2p_1/2_ are related to Ti^4+^ (TiO_2_), and peak shifts at approximately 456.3 eV and 462.2 eV are related to Ti^2+^ (TiO) [37]. As the temperature of the thermal stress increases, the peak shifts at approximately 456.3 eV and 462.2 eV increase, which proves the unstable oxidation states of Ti^2+^ (TiO) in the TiO_2_ thin film also increase [38].

**Figure 6:**
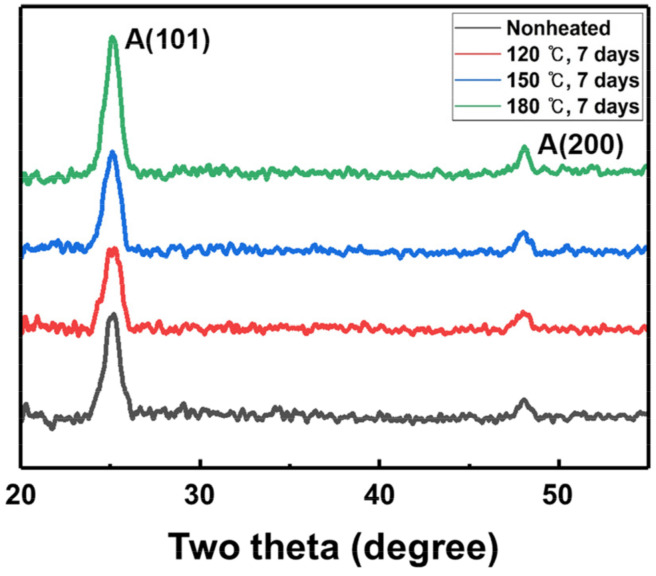
XRD spectrum of TiO_2_ thin films deposited by TTIP exposed to thermal stress.

As the temperature of the thermal stress to which TTIP was exposed increases, the crystallinity of the TiO_2_ film also increases. Figure 6 shows the XRD spectrum for the TiO_2_ film deposited by TTIP exposed to thermal stress. The XRD peak at approximately 25° is related to the anatase phase of TiO_2_. The full width at half maximum (FWHM) of TiO_2_ film deposited by TTIP not exposed to thermal stress is 1.036, and the FWHMs of TiO_2_ film deposited by TTIP exposed to thermal stresses of 120 °C, 150 °C, and 180 °C are 1.058, 1.037, and 0.985, respectively. The FWHM of the TiO_2_ film deposited with TTIP subjected to thermal stress of 180 °C decreases compared with the TiO_2_ film deposited by TTIP not exposed to thermal stress and exposed to thermal stress of 120 °C and 150 °C. Therefore, it was confirmed that the crystallinity of the TiO_2_ film deposited by TTIP exposed to thermal stress of 180 °C increases, which is a result of heavy molecular impurities generated by exposure to thermal stress acting as the crystal nucleus for thin film growth.

**Figure 7:**
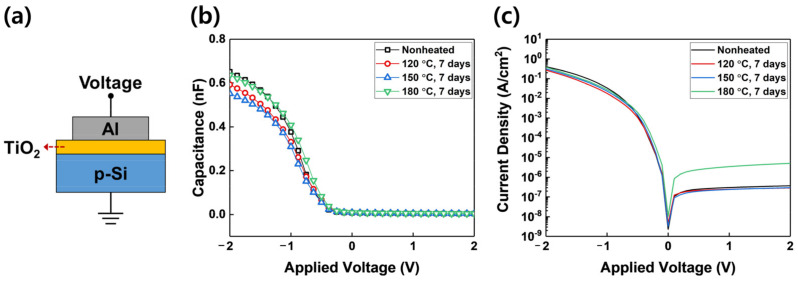
(**a**) Schematic of the connection to perform the C-V and I-V measurement, (**b**) C-V characteristics, and (**c**) I-V characteristics of the Al/TiO_2_/p-Si MOS capacitor that employed 500 cycles of TiO_2_ thin films deposited by TTIP exposed to thermal stress.

The decomposition of the precursor not only changes the thermal properties of the precursor, but also changes the thin film properties and consequently affects the properties of the device. Figure 7 shows the schematic diagram of the MOS capacitor structure and connection for performing C-V and I-V measurements and the results of the measurements that employed a TiO_2_ thin film deposited by TTIP exposed to thermal stress. The k-value of TiO_2_ film deposited by TTIP not exposed to thermal stress is 29, and the k-values of TiO_2_ film deposited by TTIP exposed to thermal stress of 120 °C, 150 °C, and 180 °C are 21, 24, and 31, respectively. The k-value decreases for the TiO_2_ films deposited by TTIP exposed to 120 °C and 150 °C thermal stress. The TiO_2_ film deposited by TTIP exposed to thermal stress of 180 °C, however, has a relatively high k-value, which is due to the influence of the thickness of the thin film. In the I-V curve, the asymmetry of the curve is a result of the interface difference on both sides of the TiO_2_ film [39], and both the MOS capacitor that employed TiO_2_ film deposited by TTIP not exposed to thermal stress and TiO_2_ film deposited by TTIP exposed to 120 °C and 150 °C show similar curves. However, the measured leakage current of the MOS capacitor that employed TiO_2_ film deposited by TTIP exposed to 180 °C is higher than other MOS capacitors. As mentioned above, this result is because of the generation of heavy molecular impurities in the film due to the decomposition of the precursor. These impurities contain carbon and can act as a leakage path [40]. In addition, impurities can form a trap site in the film and affect the reliability of the device [41]. Therefore, the results of this study suggest that the deterioration of TTIP causes not only changes in TTIP properties but also changes in device characteristics. Thus, the strict management of precursors is required for successful TiO_2_ film application.

## 4. Conclusions

This study investigated the effects of impurities and changes in physical properties when TTIP, a representative precursor for depositing TiO_2_ films, was exposed to thermal stress at different temperatures. As the temperature to which TTIP is exposed increases from 120 °C to 180 °C, the major impurities in TTIP change from light molecule impurities to heavy molecule impurities. Light molecule impurities in TTIP increase the vapor pressure but decrease the volatility of molecules effective for the growth of TiO_2_ thin films, thereby lowering the thin film growth rate. Heavy molecule impurities in TTIP reduce the vapor pressure but also act as nucleation sites of the thin film, increasing its growth rate and crystallinity. However, the unstable oxidation states of Ti^2+^ (TiO) in the thin film grow. TTIP has different behavior during the ALD process according to the characteristics of the impurities generated, and it can be confirmed that the reliability of the TiO_2_ film is degraded by changing the characteristics of the thin film and the device. ALD has recently been applied to various fields not only in semiconductors but also in display and secondary cells. Therefore, these findings are expected to provide useful information in the selection of promising precursors, process optimization, and the storage of precursors in various fields.

## Figures and Tables

**Table 1:** Antoine equation parameters of TTIP exposed to thermal stress.

	Antoine Equation Parameters		Vaporization Enthalpy ∆H (kJ/mol)
	A	B	
Nonheated	32.659	9449.569	78.6
120 °C, 7 days	33.223	9626.489	80.0
150 °C, 7 days	30.784	8844.900	73.5
180 °C, 7 days	33.052	9609.026	79.9

## Data Availability

Not applicable.

## Supplementary Materials

The following supporting information can be downloaded at: https://www.mdpi.com/article/10.3390/ma15093021/s1, Table S1: Experimental data on vapor pressure of TTIP exposed to 120~180 °C for 7 days.

## References

[1] Atanassova E., Spasov D. (2002). Thermal Ta_2_O_5_—Alternative to SiO_2_ for storage capacitor application. Microelectron. Reliab..

[2] Yu T., Jin C.G., Dong Y.J., Cao D., Zhuge L.J., Wu X.M. (2013). Temperature dependence of electrical properties for MOS capacitor with HfO_2_/SiO_2_ gate dielectric stack. Mater. Sci. Semicond. Process..

[3] Hourdakis E., Nassiopoulou A.G., Casanova A., Larrieu G. In Model 3D MOS capacitor system using regular arrays of vertical Si nanowires. Proceedings of the 2017 Joint International EUROSOI Workshop and International Conference on Ultimate Integration on Silicon (EUROSOI-ULIS).

[4] Singh R., Paily R., DasGupta A., DasGupta N., Misra P., Kukreja L.M. (2004). Optimized dual temperature pulsed laser deposition of TiO_2_ to realize MTOS (metal-TiO_2_–SiO_2_–Si) capacitors with ultrathin gate dielectric. Semicond. Sci. Technol..

[5] Houssa M., Mertens P.W., Heyns M.M. (1999). Relation between stress-induced leakage current and time-dependent dielectric breakdown in ultra-thin gate oxides. Semicond. Sci. Technol..

[6] Xu Z., Houssa M., De Gendt S., Heyns M. (2002). Polarity effect on the temperature dependence of leakage current through HfO_2_/SiO_2_ gate dielectric stacks. Appl. Phys. Lett..

[7] Park S.-U., Kang C.-Y., Kwon H.-M., Park B.-S., Choi W.-H., Han I.-S., Bersuker G., Jammy R., Lee H.-D. (2011). Analysis of reliability characteristics of high capacitance density MIM capacitors with SiO_2_–HfO_2_–SiO_2_ dielectrics. Microelectron. Eng..

[8] Jakschik S., Avellan A., Schroeder U., Bartha J.W. (2004). Influence of Al_2_O_3_ dielectrics on the trap-depth profiles in MOS devices investigated by the charge-pumping method. IEEE Trans. Electron. Devices.

[9] Nandi S.K., Chakraborty S., Bera M.K., Maiti C.K. (2007). Structural and optical properties of ZnO films grown on silicon and their applications in MOS devices in conjunction with ZrO_2_ as a gate dielectric. Bull. Mater. Sci..

[10] Zhu J., Liu Z.G. (2004). Structure and dielectric properties of ultra-thin ZrO_2_ films for high-k gate dielectric application prepared by pulsed laser deposition. Appl. Phys. A.

[11] Yang B.L., Wong H., Kakushima K., Iwai H. (2012). Improving the electrical characteristics of MOS transistors with CeO_2_/La_2_O_3_ stacked gate dielectric. Microelectron. Reliab..

[12] Lo G.Q., Kwong D.L., Lee S. (1993). Reliability characteristics of metal-oxide-semiconductor capacitors with chemical vapor deposited Ta_2_O_5_ gate dielectrics. Appl. Phys. Lett..

[13] Novkovski N. (2014). Physical modeling of electrical and dielectric properties of high-k Ta_2_O_5_ based MOS capacitors on silicon. FU Elec. Energ..

[14] Wu J.-R., Wu Y.-H., Lin C.-C., Ou W.-Y., Wu M.-L., Chen L.-L. (2012). Effect of Nitrogen Passivation on the Performance of MIM Capacitors with a Crystalline TiO_2_/SiO_2_ Stacked Insulator. IEEE Electron Device Lett..

[15] Paily R., DasGupta A., DasGupta N., Bhattacharya P., Misra P., Ganguli T., Kukreja L.M., Balamurugan A.K., Rajagopalan S., Tyagi A.K. (2002). Pulsed laser deposition of TiO_2_ for MOS gate dielectric. Appl. Surf. Sci..

[16] Chandra Sekhar M., Kondaiah P., Jagadeesh Chandra S.V., Mohan Rao G., Uthanna S. (2012). Substrate temperature influenced physical properties of silicon MOS devices with TiO_2_ gate dielectric. Surf. Interface Anal..

[17] Ahmad T., Shahazad M., Ubaidullah M., Ahmed J. (2018). Synthesis, characterization and dielectric properties of TiO_2_–CeO_2_ ceramic nanocomposites at low titania concentration. Bull. Mater. Sci..

[18] Saha D., Ajimsha R.S., Rajiv K., Mukherjee C., Gupta M., Misra P., Kukreja L.M. (2014). Spectroscopic ellipsometry characterization of amorphous and crystalline TiO_2_ thin films grown by atomic layer deposition at different temperatures. Appl. Surf. Sci..

[19] Triyoso D.H., Hegde R.I., Grant J., Fejes P., Liu R., Roan D., Ramon M., Werho D., Rai R., La L.B. (2004). Film properties of ALD HfO_2_ and La_2_O_3_ gate dielectrics grown on Si with various pre-deposition treatments. J. Vac. Sci. Technol..

[20] Niemelä J.-P., Marin G., Karppinen M. (2017). Titanium dioxide thin films by atomic layer deposition: A review. Semicond. Sci. Technol..

[21] Leskelä M., Ritala M. (2002). Atomic layer deposition (ALD): From precursors to thin film structures. Thin Solid Film..

[22] Zydor A., Elliott S.D. (2010). Thermal Stability of Precursors for Atomic Layer Deposition of TiO_2_, ZrO_2_, and HfO_2_: An Ab Initio Study of α-Hydrogen Abstraction in Bis-Cyclopentadienyl Dimethyl Complexes. J. Phys. Chem. A.

[23] Blanquart T., Niinistö J., Gavagnin M., Longo V., Pallem V.R., Dussarrat C., Ritala M., Leskelä M. (2012). Novel heteroleptic precursors for atomic layer deposition of TiO_2_. Chem. Mater..

[24] Niinistö J., Mäntymäki M., Kukli K., Costelle L., Puukilainen E., Ritala M., Leskelä M. (2010). Growth and phase stabilization of HfO_2_ thin films by ALD using novel precursors. J. Cryst. Growth.

[25] Rushworth S., Coward K., Davies H., Heys P., Leese T., Kempster L., Odedra R., Song F., Williams P. (2007). Thermal stability studies for advanced Hafnium and Zirconium ALD precursors. Surf. Coat. Technol..

[26] Lee B., Choi K.J., Hande A., Kim M.J., Wallace R.M., Kim J., Senzaki Y., Shenai D., Li H., Rousseau M. (2009). A novel thermally-stable zirconium amidinate ALD precursor for ZrO_2_ thin films. Microelectron. Eng..

[27] Niinistö J., Kukli K., Kariniemi M., Ritala M., Leskelä M., Blasco N., Pinchart A., Lachaud C., Laaroussi N., Wang Z. (2008). Novel mixed alkylamido-cyclopentadienyl precursors for ALD of ZrO_2_ thin films. J. Mater. Chem..

[28] Pinchart A., Blasco N., Lachaud C., Schleisman A., Dussarrat C., Suzuki I., Yanagita K. Novel thermally-stable hafnium and zirconium ALD precursors. Proceedings of the 2007 IEEE/SEMI Advanced Semiconductor Manufacturing Conference.

[29] Blanquart T., Niinisto J., Aslam N., Banerjee M., Tomczak Y., Gavagnin M., Longo V., Puukilainen E., Wanzenboeck H.D., Kessels W.M.M. (2013). [Zr(NEtMe)_2_(guan-NEtMe)_2_] as a novel atomic layer deposition precursor: ZrO_2_ film growth and mechanistic studies. Chem. Mater..

[30] Niinistö J., Rahtu A., Putkonen M., Ritala M., Leskelä M., Niinistö L. (2005). In Situ Quadrupole Mass Spectrometry Study of Atomic-Layer Deposition of ZrO_2_ using Cp_2_Zr(CH_3_)_2_ and water. Lanmuir.

[31] Aarik J., Aidla A., Uustare T., Ritala M., Leskelä M. (2000). Titanium isopropoxide as a precursor for atomic layer deposition: Characterization of titanium dioxide growth process. Appl. Surf. Sci..

[32] Yun J.-Y., Heo S.-W., Kang S.-W., Na J.-G., Park Y.-J., Shin Y.-H., Lee J.-H., Kim T.-S., Moon D.-K. (2008). A study on the real-time decomposition monitoring of a metal organic precursor for metal organic chemical vapor deposition processes. Meas. Sci. Technol..

[33] Soulet A., Duquesne L., Jursich G., Inman R., Misra A., Blasco N., Lachaud C., Marot Y., Prunier R., Vautier M. (2005). Optimizing the selection and supply of Hf precursor candidates for gate oxide. Semicond. Fabtech.

[34] Wang C., Yang S., Chen Y. (2019). Determination of the vapour pressure curves and vaporization enthalpies of hafnium alkoxides using thermogravimetric analysis. R. Soc. Open Sci..

[35] Blackburn B.J., Drosos C., Brett D.B., Parkes M.A., Carmalt C.J., Parkin I.P. (2016). In situ mass spectrometry analysis of chemical vapour deposition of TiO_2_ thin films to study gas phase mechanisms. RSC Adv..

[36] Determination of Sublimation Enthalpy and Vapor Pressure for Inorganic and Metal-Organic Compounds by Thermogravimetric Analysis. http://cnx.org/content/m33649/1.2/.

[37] Hannula M., Ali-Löytty H., Lahtonen K., Sarlin E., Saari J., Valden M. (2018). Improved Stability of Atomic Layer Deposited Amorphous TiO_2_ Photoelectrode Coatings by Thermally Induced Oxygen Defects. Chem. Mater..

[38] Zafar M., Yun J.-Y., Kim D.-H. (2018). Performance of inverted organic photovoltaic cells with nitrogen doped TiO_2_ films by atomic layer deposition. Korean J. Chem. Eng..

[39] Kumar A., Mondal S., Rao K.K. (2016). Low temperature solution processed high-κ ZrO_2_ gate dielectrics for nanoelectonics. Appl. Surf. Sci..

[40] Jin H.S., Kim D.H., Kim S.K., Wallace R.M., Kim J., Park T.J. (2019). Strategic Selection of the Oxygen Source for Low Temperature-Atomic Layer Deposition of Al_2_O_3_ Thin Film. Adv. Electron. Mater..

[41] Shibata T., Uenuma M., Yamada T., Yoshitsugu K., Higashi M., Nishimura K., Uraoka Y. (2022). Effects of carbon impurity in ALD-Al_2_O_3_ film on HAXPES spectrum and electrical properties of Al_2_O_3_/AlGaN/GaN MIS structure. Jpn. J. Appl. Phys..

