# Supplementary material for: Decomposition Characteristics of the TTIP (Tetraisopropyl Orthotitanate) Precursor for Atomic Layer Deposition
Source: Materials (Basel). 2022 Apr 21;15(9):3021. doi: 10.3390/ma15093021 (PMC9100524; doi:10.3390/ma15093021)
Supplement: Supplementary file 1 [file materials-15-03021-s001.zip › materials-1670932-supplementary.pdf]

Table S1. Experimental data on vapor pressure of TTIP exposed to 120~180 °C for 7 days.

	Nonheated TTIP			TTIP exposed to 120 °C for 7 days		
	**Value**	**Average**	**Std. Dev.**	**Value**	**Average**	**Std. Dev.**
**318.15 K**	18.268			19.019		
**318.15 K**	19.043			18.653		
**318.15 K**	18.654	18.818	0.399	19.295	19.090	0.388
**318.15 K**	18.803			18.843		
**318.15 K**	19.323			19.641		
**323.15 K**	31.455			31.604		
**323.15 K**	32.045			32.713		
**323.15 K**	31.417	31.960	0.567	32.191	32.075	0.503
**323.15 K**	32.076			32.342		
**323.15 K**	32.807			31.528		
**328.15 K**	47.599			48.382		
**328.15 K**	47.004			47.093		
**328.15 K**	47.241	46.501	1.512	47.233	47.973	0.748
**328.15 K**	46.816			48.671		
**328.15 K**	43.848			48.484		

	TTIP exposed to 150 °C for 7 days			TTIP exposed to 180 °C for 7 days		
	**Value**	**Average**	**Std. Dev.**	**Value**	**Average**	**Std. Dev.**
**318.15 K**	19.335			17.753		
**318.15 K**	18.406			17.673		
**318.15 K**	18.621	19.148	0.604	17.699	17.631	0.052
**318.15 K**	19.615			17.629		
**318.15 K**	19.764			17.402		
**323.15 K**	32.757			27.188		
**323.15 K**	32.387			26.311		
**323.15 K**	31.369	32.359	0.574	26.800	26.469	0.625
**323.15 K**	32.543			25.518		
**323.15 K**	32.740			26.528		
**328.15 K**	45.066			44.577		
**328.15 K**	45.301			43.878		
**328.15 K**	44.275	44.616	0.549	43.900	44.287	0.364
**328.15 K**	44.443			44.526		
**328.15 K**	43.995			44.556		
